# Validation of the healthcare workers’ version of the Pandemic Grief Scale among frontline nursing professionals during the COVID-19 pandemic in Korea

**DOI:** 10.3389/fpsyt.2023.1121546

**Published:** 2023-03-30

**Authors:** Jeong Hye Kim, C. Hyung Keun Park, Oli Ahmed, Youjin Hong, Seockhoon Chung, Jangho Park, Sherman A. Lee

**Affiliations:** ^1^Department of Clinical Nursing, University of Ulsan, Seoul, Republic of Korea; ^2^Department of Psychiatry, ASAN Medical Center, University of Ulsan College of Medicine, Seoul, Republic of Korea; ^3^Department of Psychology, University of Chittagong, Chattogram, Bangladesh; ^4^National Centre for Epidemiology and Population Health, Australian National University, Canberra, ACT, Australia; ^5^Department of Psychiatry, GangNeung Asan Hospital, University of Ulsan College of Medicine, Gangneung, Republic of Korea; ^6^Department of Psychiatry, Ulsan University Hospital, University of Ulsan College of Medicine, Ulsan, Republic of Korea; ^7^Department of Psychology, Christopher Newport University, Newport News, VA, United States

**Keywords:** COVID-19, pandemics, grief, health personnel, nurses

## Abstract

**Introduction:**

Nurses have been repeatedly exposed to unexpected death and grief during COVID-19 pandemic, and it is necessary to provide grief support for the nurses who have experienced the loss of patients to COVID-19. We aimed to explore the reliability and validity of the Pandemic Grief Scale (PGS) for Healthcare Workers among frontline nursing professionals working in COVID-19 inpatient wards whose patients may have died.

**Methods:**

An anonymous online survey was performed among frontline nursing professionals working in COVID-19 wards in three tertiary-level general hospitals in Korea between April 7 and 26, 2021. In total, 229 from participants who confirmed they had witnessed death of patients were employed for the statistical analysis. The survey included demographic characteristics and rating scales, including the Korean version of the PGS for Healthcare Workers, the Fear of COVID-19 scale, the Generalized Anxiety Disorder-7 items, and the Patient Health Questionnaire-9 items.

**Results:**

The single-factor structure of the Korean version of the PGS for Healthcare Workers showed good fits for the model. The scale had good internal consistency and convergent validity with other anxiety and depression scales.

**Conclusion:**

The Korean version of the PGS of Healthcare Workers was valid and reliable for measuring grief reactions among nursing professionals facing the pandemic. It will be helpful in evaluating the grief reaction of the healthcare workers and providing them with a psychological support system.

## 1. Introduction

Since the first case of the coronavirus disease 2019 (COVID-19) was identified over 2 years ago, the world has witnessed the rapid spread of SARS-CoV-2. As of April 30, 2022, 510,824,055 cases of COVID-19 were confirmed and 6,235,957 COVID-19 deaths were reported worldwide (1). South Korea, a country known for epidemic preparedness, reported 17,237,878 confirmed cases of COVID-19 and 22,794 deaths on that same day (2). Although the main focus is on the physical health consequences of an infectious disease outbreak, rates of mental illnesses, such as depression, anxiety, and post-traumatic stress disorder also rise during of global health emergencies (3). According to a systematic review, the prevalence of stress, anxiety, and depression in the population during the COVID-19 pandemic is alarming, with rates as high as 29.6%, 31.9%, and 33.7%, respectively (4). With complete eradication of SARS-CoV-2 appearing unrealistic, the Korean government implemented the policy of coexistence with COVID-19 in November, 2021, which loosened restrictions. Shortly after the implementation of this policy, as somewhat expected, the number of confirmed COVID-19 cases increased rapidly, reaching 621,328 daily cases in only 4 months (2).

One of the groups most severely impacted by the COVID-19 pandemic are healthcare workers. They work long hours in the frontline of the pandemic, diagnosing and treating patients while being continuously exposed to their patients’ pain and suffering. This unprecedented situation has not only entailed high levels of physical exhaustion for healthcare workers but has also caused severe psychological conditions, such as depression, anxiety, and insomnia (5). A systematic review of 38 studies on the mental health problems of healthcare workers (e.g., doctors, nurses, and allied health workers) during the COVID-19 pandemic revealed that the estimated prevalence of depression, anxiety, and post-traumatic stress disorder is higher than thatof the general population, with estimates of 37% (95% confidential interval [CI]: 29%–45%), 40% (95% CI: 29%–52%), and 49% (95% CI: 25%–50%) (6), respectively. Unsurprisingly, many of these healthcare workers reported fear of contracting and transmitting the virus (6) while maintaining an unusually high and stressful workload (7).

Nurses play a unique and important role in a healthcare system by helping patients and their families cope with death and grief (8). Moreover, nurses commonly witness multiple patient deaths within a brief period. Although their invaluable services entail numerous benefits, their exposure to such traumatic experiences adversely affects their psychological and personal well-being over time (9). Repeated exposure to death and grief can result in occupational stress and burnout in addition to the emotional distress nurses encounter (10). During the COVID-19 pandemic, social distancing policies restricted in-person contact between patients and their loved ones. End-of-life COVID-19 patients who were quarantined were not allowed to visit their immediate family members (8). In this context, nurses also played the intimate role of caregiver and emotional support provider for COVID-19 patients, some of which died in isolation without loved ones and family around (8). Some nurses described this situation as a “tsunami of death,” reporting uncharacteristically high levels of psychological distress caused by witnessing patient deaths due to COVID-19 (11). Given the pandemic situation and the important role nurses play in the healthcare system, grief support must be provided for the nurses who have experienced patient loss due to COVID-19.

During the pandemic, the Pandemic Grief Scale (PGS) (12) was developed to help clinicians and researchers effectively identify individuals suffering from dysfunctional levels of grief due to COVID-19 deaths. Since its publication, the PGS has been validated in different countries and effectively used in various settings (12–14). However, the validation of a healthcare version of this instrument has yet to be published. Therefore, we aimed to explore the reliability and validity of the PGS for healthcare workers using frontline nursing professionals who worked with COVID-19 inpatients and witnessed the death of their patients.

## 2. Method

### 2.1. Participants

An online survey was conducted among frontline nursing professionals working in COVID-19 inpatient wards at three tertiary-level affiliated hospitals of the University of Ulsan, including the Asan Medical Center in Seoul, the Ulsan University Hospital in Ulsan, and the GangNeung Asan Hospital in Gangneung, from April 7 to April 26, 2022. All participants participated voluntarily and were rewarded with a gift coupon worth 10 US dollars for their participation. No personal information was collected. This survey study was approved by the Institutional Review Board of the Asan Medical Center (2022-0323), the Ulsan University Hospital (UUH 2022-02-016-003), and the GangNeung Asan Hospital (2022-03-003-001), and written informed consent was waived for study participation. In the survey, participants’ age, sex, years of employment, work shifts, and marital status were obtained. They were also asked to respond to questions about COVID-19, such as whether they are currently caring for infected patients or have been quarantined, infected, or vaccinated, their past psychiatric history and their current psychiatric distress. The survey was designed according to CHERRIES guidelines (15), and the usability and technical functionality of the survey was tested by the principal investigator (SC) before it was implemented.

All 439 nursing professionals (239 from the Asan Medical Center, 150 from the Ulsan University Hospital, and 50 from the GangNeung Asan Hospital) working in COVID-19 inpatients wards were considered in the sample size estimation. Data were collected from at least 60% (*N* = 203) of the eligible patient population. The sample size was 229; there were 126, 85, and 18 responses of participants who confirmed that they witnessed patient death from each hospital.

### 2.2. Symptom assessment

#### 2.2.1. Pandemic Grief Scale for healthcare workers

The PGS was originally designed to assess grief reactions to COVID-19 (12). Investigator Dr. Sherman Lee revised the original scale into a version intended for healthcare workers (Supplementary Table 1). This tool is designed to screen for dysfunctional grief following a patient loss due to COVID-19. This PGS was developed using a large sample size of adults (*N* = 831) who lost someone significant to them to the virus. The PGS comprises five items rated on a 4-point scale, ranging from a score of 0 (not at all) to 3 (nearly every day), on the basis of experiences over the past 2 weeks. A total PGS score of seven indicates probable dysfunctional grief due to a COVID-19 loss. High scores on a particular item or a high total scale score (≥7) may indicate problematic symptoms that require further assessment and/or treatment. By using translation and back-translation methods, a bilingual expert translated the original English version of the PGS for healthcare workers into a Korean version. A second bilingual expert translated all the new Korean text back into English without any reference to the original text. A third party compared and verified the original English version and the reversed translated English version and found subtle variations. Following these steps, the Korean version of the PGS for healthcare workers was completed. The content of the scale was not changed or added. Permission was obtained from the developer of the PGS to translate the scale.

#### 2.2.2. Fear of COVID-19 scale

The FCV-19S is a self-reported rating scale for quantifying one’s viral anxiety during the COVID-19 pandemic (16). There are 7 items in the FCV-19S, each rated from 1 (strongly disagree) to 5 (strongly agree). A higher total score indicates a higher level of viral anxiety. For this study, the Korean form of the FCV-19S was used (17). Cronbach’ alpha was 0.873 among this sample.

#### 2.2.3. Generalized Anxiety Disorder-7 items (GAD-7)

The GAD-7 is a self-report scale used to assess the severity of general anxiety (18). It consists of seven items rated from zero (never at all) to three (nearly every day). An elevated total score indicates a severe level of anxiety. The Koran version of the GAD-7 (19) was used in this study, and Cronbach’s alpha was 0.880 in this sample.

#### 2.2.4. Patient Health Questionnaire-9 items (PHQ-9)

The PHQ-9 is a self-rating scale that measures depression severity (20). It adopts 9 items rated on a Likert scale from 0 (not at all) to 3 (nearly every day). The higher the total score, the more severe the depression. Our study used the Korean version of PHQ-9 (19), and Cronbach’s alpha was 0.934.

### 2.3. Statistical analysis

The factor structure of the scale utilizing confirmatory factor analysis (CFA) was assessed through the Diagonally Weighted Least Squares (DWLS) estimation method. To check the sampling adequacy and data suitability for the factor analyses, Kaiser-Meyer-Olkin (KMO) value and Bartlett’s test of sphericity were examined prior to run CFA. In the CFA, satisfactory model fit was defined by a standardized root-mean-square residual (SRMR) value ≤0.05, RMSEA value ≤0.10, and comparative fit index (CFI) and goodness of fit index (GFI) values ≥0.90 (21, 22). Multi-group CFA was to assess the measurement invariance across being afraid of COVID-19 (FCV-19S ≥ 17), having depression (PHQ-9 ≥ 10), and having anxiety (GAD-7 ≥ 10). The psychometric properties of this scale was also assessed using the Item Response Theory (IRT) Approach through Graded Response Model (GRM). Before running the GRM, IRT assumptions — unidimensionality (Loevinger’s *H* coefficients), local dependence (*p*-values [adjusted for false discovery rate] of *G*^2^) and monotonicity (the number of significant violations and *Crit* value) were evaluated. In the GRM, item fits through S-*χ*^2^ and its *p*-values (adjusted for false discovery rate) and RMSEA (≤ 0.10) were assessed. Next, the slope parameters (*α*) and threshold parameters (*b*) of the items were assessed and the scale information curve of the PGS scale was extracted. Reliability test of the PGS was performed using Cronbach’s alpha, McDonald’s Omega, and split-half reliability (odd-even). The SPSS version 21.0, RStudio, and jMetrik softwares were used for statistical analysis.

## 3. Results

A total of 229 respondents (94.3% women, 5.7% men) participated in the study (Table 1). The mean age was 30.1 (±6.3) years, with the majority (175, 76.4%) being single. The average years of employment were 6.9 (±6.0) years, and 218 (95.2%) worked on a shift basis. All respondents were caring for COVID-19 infected patients at the time of the survey and had witnessed patient deaths. All were fully vaccinated, with 95 (41.5%) and 85 (37.1%) having been quarantined and infected, respectively, due to COVID-19. Regarding psychiatric problems, 37 (16.2%) reported a history of depression, anxiety, or insomnia, and 33 (14.4%) complained of present depression or anxiety.

**Table 1:** Clinical characteristics of participants (*N* = 229).

Variables	*N* (%) Mean ± SD
Sex (female)	216 (94.3%)
Age	30.1 ± 6.3
Years of employment	6.9 ± 6.0
Marital status^a^	
Single	175 (76.4%)
Married, without kids	17 (7.4%)
Married, with kids	35 (15.3%)
Are you a shift worker?	218 (95.2%)
Questions on COVID-19	
Are you taking care of COVID-19 infected patients? (Yes)	229 (100.0%)
Did you experience being quarantined due to infection with COVID-19? (Yes)	95 (41.5%)
Have you experienced being infected with COVID-19? (Yes)	85 (37.1%)
Did you get vaccinated? (Yes)	136 (100.0%)
Have you experienced deaths of COVID-19 infected patients? (Yes)	100 (100.0%)
Psychiatric history	
Did you experience or treat depression, anxiety, or insomnia? (Yes)	37 (16.2%)
Do you think you are depressed or anxious now or that you need help for your mood state? (Yes)	33 (14.4%)
Rating scales scores	
Pandemic Grief Scale	1.4 ± 2.9
Fear of COVID-19 scale	17.0 ± 5.4
Patient Health Questionnaire-9 items	8.1 ± 5.3
Generalized Anxiety Disorders-7 items	4.2 ± 4.8

a There were two missing values.

### 3.1. Confirmatory factor analysis

Item-level properties of the PGS for healthcare workers are shown in Table 2. Based on the KMO value (0.87) and Bartlett’s test of Sphericity (*p* < 0.001, Table 3), we observed that sampling was adequate and data was suitable for conducting CFA.

**Table 2:** Item properties of the PGS for healthcare workers.

Items	Descriptive		CITC	CID	Factor loading
	*M*	SD			
1. I wished to die in order to be with all of the patients I knew who died of COVID-19.	0.082	0.381	0.720	0.844	0.782
2. I experienced confusion over my role in life or felt like my identity was diminished because of all of the patients I knew who died of COVID-19.	0.267	0.571	0.701	0.834	0.763
3. Nothing seemed to matter much to me because of all of the patients I knew who died of COVID-19.	0.306	0.615	0.707	0.833	0.770
4. I found it difficult to have positive memories about all of the patients I knew who died of COVID-19.	0.341	0.671	0.662	0.850	0.718
5. I believed that without all of the patients I knew who died of COVID-19, life was either meaningless, empty, or could not go on.	0.198	0.546	0.734	0.826	0.802

M, Mean; SD, Standard deviation; CITC, Corrected item-total correlation; CID, Cronbach’s alpha if item deleted; CFA, Confirmatory factor analysis.

**Table 3:** Scale-level psychometric properties of the PGS for healthcare workers.

Psychometric properties	Scores	Suggested cut off
Internal consistency reliability		
Cronbach’s alpha	0.866	≥ 0.7
McDonald’s Omega	0.870	≥ 0.7
Split-half reliability (odd-even)	0.858	≥ 0.7
Composite reliability	0.877	≥ 0.7
Standard error of measurement	0.834	Smaller than SD (2.279)/2
Statistics from exploratory factor analysis		
KMO measure of sample adequacy	0.87	0.50
Bartlett’s test of sphericity	548.5745 (<0.001)	Significant
Model fits of confirmatory factor analysis		
*χ*^2^ (df, *p* value)	0.124 (5, 1.000)	Nonsignificant
CFI	1.000	>0.95
TLI	0.999	>0.95
RMSEA	0.000	<0.08
SRMR	0.019	<0.08

SD, Standard deviation; KMO, Kaiser-Meyer-Olkin; CFI, Comparative fit index; TLI, Tucker–Lewis index; RMSEA, Root mean square error of approximation; SRMR, Standardized root-mean-square residual.

CFA results indicated good fit for single factor model of the PGS for healthcare workers (*χ^2^* = 0.124, df = 5, *p* value = 1.000, CFI = 1.000, TLI = 0.999, RMSEA = 0.000, SRMR = 0.019) (Table 3). Factor loadings ranged between 0.718 and 0.802 (Table 2). Multi-group CFA results demonstrated that the PGS for healthcare workers can assess healthcare workers’ grief in a same way across having viral anxiety (FCV-19S > 17), having depression (PHQ-9 ≥ 10), or having general anxiety (Supplementary Table 2).

### 3.2. Graded response model analysis

Table 3 and Supplementary Table 3 present the information on IRT assumptions. Loevinger’s *H* coefficient (0.678) suggests the PGS is highly unidimensional (Table 3). Non-significant *p*-values (adjusted for false discovery rate) suggest the absence of possible local dependence (Supplementary Table 3). Absence of significant violation and zero crit values for items indicate that the monotonicity assumption meets. Regarding item fits, non-significant p-values (adjusted for false discovery rate) and RMSEA values suggest that all the items belong to the same latent construct (Supplementary Table 4). Regarding slope parameters, all the items of the PGS exhibit a very high slope coefficient, ranging from 2.416 to 5.948 (mean = 3.565) (Supplementary Table 4). All these items are highly efficient and able to provide reliable information about the latent trait assessed by the Korean version of the PGS. Regarding threshold parameters, an above average level of latent trait is required to endorse all the Likert-type response options in all items. Scale information curve (Supplementary Figure 1) shows that the Korean version of the PGS provides more information about people between 0.3 and 3.3 θ level.

### 3.3. Reliability of the PGS for healthcare worker and evidence based on relations to other variables

Item analysis results show that all items have acceptable corrected item-total correlation, ranging between 0.662 and 0.734 (Table 2). The Korean version of the PGS for healthcare workers has high internal consistency reliability (Cronbach’s alpha = 0.866, McDonald’s omega = 0.870, and Split-half reliability = 0.858). This scale also has good composite reliability (0.877). The standard error of measurement (0.834) was below the cut off (smaller than SD (2.279)/2). The PGS for healthcare worker showed a good convergent validity with FCV-19S (*r* = 0.412, 95% CI [0.514, 0.299], *p* < 0.001), PHQ-9 (*r* = 0.469, 95% CI [0.565, 0.362], *p* < 0.001), and GAD-7 (*r* = 0.589, 95% CI [0.667, 0.497], *p* < 0.001). PGS total score was significantly higher for having viral anxiety (FCV-19S > 17, *t*(227) = 3.604, *p* < 0.001), having depression (PHQ-9 ≥ 10, *t*(227) = 4.773, *p* < 0.001), or having general anxiety (GAD-7 ≥ 10, *t*(227) = 8.633, *p* < 0.001).

## 4. Discussion

The present study is the first to explore the reliability and validity of the PGS for healthcare workers, which measures healthcare workers’ dysfunctional grief over the death of patients caused by COVID-19. The results of this study provide ample evidence for the psychometric integrity of this PGS for healthcare workers.

Specifically, we observed that the PGS for healthcare workers showed good internal consistency and reliability and yielded acceptable corrected item-total correlation, as well as good composite reliability or standard error of measurement. This model also demonstrated good convergent validity with rating scales for measuring viral anxiety, depression, or generalized anxiety. Regarding factor analysis results, this version of the PGS indicated good model fit for a single factor model, with item factor loadings ranging between 0.718 and 0.802. As this is the first validation study of the PGS for healthcare workers, no comparable studies exist. From the multi-group CFA, we observed that the PGS for healthcare workers can measure dysfunctional grief responses similarly across nurses with viral anxiety, depression, or general anxiety. Regarding slope parameters, all items of the PGS have a very high slope coefficient, ranging from 2.416 to 5.948 (mean = 3.565) (Supplementary Table 4). In terms of threshold parameters, an above average level of latent trait is required to endorse all the Likert-type response options in all items. Scale information curve (Supplementary Figure 1) demonstrates that this version of the PGS provides more information about people between 0.3 and 3.3 θ level. Collectively, these results suggest that all the items of this scale are highly efficient and provide useful information about the latent trait.

The need to develop and validate the PGS for healthcare workers is based on two important factors: First, although the number of infected people worldwide has been decreasing recently (1), the disappearance of the virus seems unlikely as new variants continue to be detected. Second, grief caused by COVID-19-related losses has been found in some studies to be more intense than deaths from other causes (23). Thus, the need for a short screening instrument that can identify individuals experiencing dysfunctional grief due to COVID-19 deaths appears vital during this pandemic. Third, nurses play a vital role in any healthcare system, particularly during a pandemic. However, the emotional toll of working with patients who die of an infectious disease, such as COVID-19, is particularly high for healthcare professionals (5). Nurses experience a range of emotional responses, such as sadness, helplessness, loss, and guilt, when a patient they work with dies (24). They not only grieve over the loss of patients but also feel guilty about their patients’ deaths given that they consider these losses as failures of their medical treatment (25). Moreover, nurses may develop compassion fatigue, emotional exhaustion, and insomnia when they witness numerous patient deaths (26) that are coupled with the common excessive workload during this COVID-19 pandemic. Given these psychological issues, we believe a healthcare-focused version of the PGS is warranted.

This study holds several limitations. First, it was aimed at those who witnessed patient death among frontline nurses in a COVID-19 inpatient ward. However, healthcare workers who have experienced patient losses due to COVID-19 are not limited to healthcare workers in dedicated wards. Although it depends on the characteristics of the hospital, for patients who died in the general ward, the exact cause of death may not be known and determining whether they died from COVID-19 may be difficult. Therefore, the reliability and validity of the PGS scale must be expanded to encompass not just healthcare workers in COVID-19 dedicated wards but also other healthcare workers who witnessed patient deaths during the pandemic. This aspect must be addressed in future studies. Second, only nurses participated in our study. Despite the fact that nurses’ depression, general anxiety, and virus-related anxiety symptoms are among the highest across healthcare professions during the COVID-19 pandemic (27), future research would benefit from including other healthcare professionals. Finally, the adaptation of healthcare workers to the COVID-19 situation during the second year of the pandemic may have affected the results of the study. Future research should determine if the PGS is sensitive to temporal changes. This study has a strength despite its limitations; it is the first to present a Korean-language instrument for identifying nurses experiencing dysfunctional levels of grief during the pandemic.

In conclusion, we found the PGS for healthcare workers to be a psychometrically sound tool. The rating scale was valid and reliable for measuring dysfunctional grief reactions among nursing professionals facing viral epidemics. Thus, this PGS version will be beneficial to healthcare workers for a system that provides psychological support.

## Data availability statement

The raw data supporting the conclusions of this article will be made available by the authors, without undue reservation.

## Ethics statement

The studies involving human participants were reviewed and approved by Institutional Review Board of the Asan Medical Center, Institutional Review Board of Ulsan University Hospital, and Institutional Review Board of GangNeung Asan Hospital. Written informed consent for participation was not required for this study in accordance with the national legislation and the institutional requirements.

## Author contributions

CP, SC, JP, and SL contributed to conception and design of the study. OA and SC performed the statistical analysis. JK and SC wrote the first draft of the manuscript. JK, CP, YH, and JP wrote sections of the manuscript. All authors contributed to the manuscript revision, read, and approved the submitted version.

## Conflict of interest

The authors declare that the research was conducted in the absence of any commercial or financial relationships that could be construed as a potential conflict of interest.

## Publisher’s note

All claims expressed in this article are solely those of the authors and do not necessarily represent those of their affiliated organizations, or those of the publisher, the editors and the reviewers. Any product that may be evaluated in this article, or claim that may be made by its manufacturer, is not guaranteed or endorsed by the publisher.
