# Supplementary material for: Validation of the healthcare workers’ version of the Pandemic Grief Scale among frontline nursing professionals during the COVID-19 pandemic in Korea
Source: Front Psychiatry. 2023 Mar 30;14:1121546. doi: 10.3389/fpsyt.2023.1121546 (PMC10098354; doi:10.3389/fpsyt.2023.1121546)
Supplement: Supplementary file 1 [file Data_Sheet_1.docx]

**Supplementary Table 1. The original Pandemic Grief Scale and its revised version for healthcare workers**

| **Items** | **Original scales** | **Revised for healthcare workers** |
| --- | --- | --- |
| **Item 1** | I wished to die in order to be with the deceased. | I wished to die in order to be with all of the patients I knew who died of COVID-19. |
| **Item 2** | I experienced confusion over my role in life or felt like my identity was diminished because of the loss. | I experienced confusion over my role in life or felt like my identity was diminished because of all of the patients I knew who died of COVID-19. |
| **Item 3** | Nothing seemed to matter much to me because of this loss. | Nothing seemed to matter much to me because of all of the patients I knew who died of COVID-19. |
| **Item 4** | I found it difficult to have positive memories about the deceased. | I found it difficult to have positive memories about all of the patients I knew who died of COVID-19. |
| **Item 5** | I believed that without the deceased, life was either meaningless, empty, or could not go on. | I believed that without all of the patients I knew who died of COVID-19, life was either meaningless, empty, or could not go on. |

**Supplementary Table 2. Measurement invariance across viral anxiety, depression, and generalized anxiety**

| **Model** | **χ^2^** | **df** | **Δ χ^2^** | **Δdf** | **p** | **CFI** | **ΔCFI** | **RMSEA** | **ΔRMSEA** |
| --- | --- | --- | --- | --- | --- | --- | --- | --- | --- |
| **Having viral anxiety (FCV-19S > 17 vs. FCV-19S ≤ 17)** | | | | | | | | | |
| **Configural** | 2.190 | 10 |  |  |  | 1.000 |  | .000 |  |
| **Metric** | 4.842 | 14 | 2.652 | 4 | .618 | 1.000 | .000 | .000 | .000 |
| **Scalar** | 6.103 | 18 | 1.261 | 4 | .868 | 1.000 | .000 | .000 | .000 |
| **Having depression (PHQ-9 ≥ 10 vs. PHQ-9 < 10)** | | | | | | | | | |
| **Configural** | .322 | 10 |  |  |  | 1.000 |  | .000 |  |
| **Metric** | .654 | 14 | 0.332 | 4 | .988 | 1.000 | .000 | .000 | .000 |
| **Scalar** | 3.756 | 18 | 3.102 | 4 | .541 | 1.000 | .000 | .000 | .000 |
| **Having anxiety (GAD-7 ≥ 10 vs. GAD-7 < 10)** | | | | | | | | | |
| **Configural** | .790 | 10 |  |  |  | 1.000 |  | .000 |  |
| **Metric** | 5.370 | 14 | 4.580 | 4 | .333 | 1.000 | .000 | .000 | .000 |
| **Scalar** | 7.060 | 18 | 1.690 | 4 | .793 | 1.000 | .000 | .000 | .000 |

**Supplementary Table 3. Monotonicity and local dependency information of the PGS for healthcare workers**

|  | ***H* coefficients** | **Monotonicity** | | | | **Local dependance G^2^ p values** | | | |
| --- | --- | --- | --- | --- | --- | --- | --- | --- | --- |
|  |  | **#ac** | **#vi** | **#zsig** | ***Crit*** | **Item1** | **Item2** | **Item3** | **Item4** |
| **Item1** | 0.678 | 0 | 0 | 0 | 0 |  |  |  |  |
| **Item2** |  | 1 | 0 | 0 | 0 | .313 |  |  |  |
| **Item3** |  | 2 | 0 | 0 | 0 | .065 | .065 |  |  |
| **Item4** |  | 3 | 0 | 0 | 0 | .138 | .089 | .065 |  |
| **Item5** |  | 1 | 0 | 0 | 0 | .180 | .065 | .062 | .062 |
| **ac = active comparison, vi = violation, zsig = significant violation**  **Notes: p-values adjusted for false discovery rate (FDR)** | | | | | | | | | |

**Supplementary Table 4. Items fits, slope, and threshold parameters of the PGS for healthcare workers**

| **Items** | **Item fits** | | | | **Slope parameter (a)** | **Threshold parameter (b)** | | |
| --- | --- | --- | --- | --- | --- | --- | --- | --- |
|  | **S-χ^2^** | **df** | **p value** | **RMSEA** |  | **b_1_** | **b_2_** | **b_3_** |
| **Item 1** | NaN |  | NaN | NaN | 5.948 | 1.548 | 2.246 | 2.912 |
| **Item 2** | .332 | 1 | .564 | .000 | 3.470 | .870 | 1.896 | 2.689 |
| **Item 3** | 4.872 | 2 | .210 | .079 | 2.646 | .820 | 2.071 | 2.487 |
| **Item 4** | 1.879 | 2 | .521 | .000 | 2.416 | .807 | 1.871 | 2.449 |
| **Item 5** | 2.629 | 1 | .210 | .084 | 3.343 | 1.155 | 2.011 | 2.501 |
| **Notes: p-values adjusted for false discovery rate (FDR)** | | | | | | | | |

**
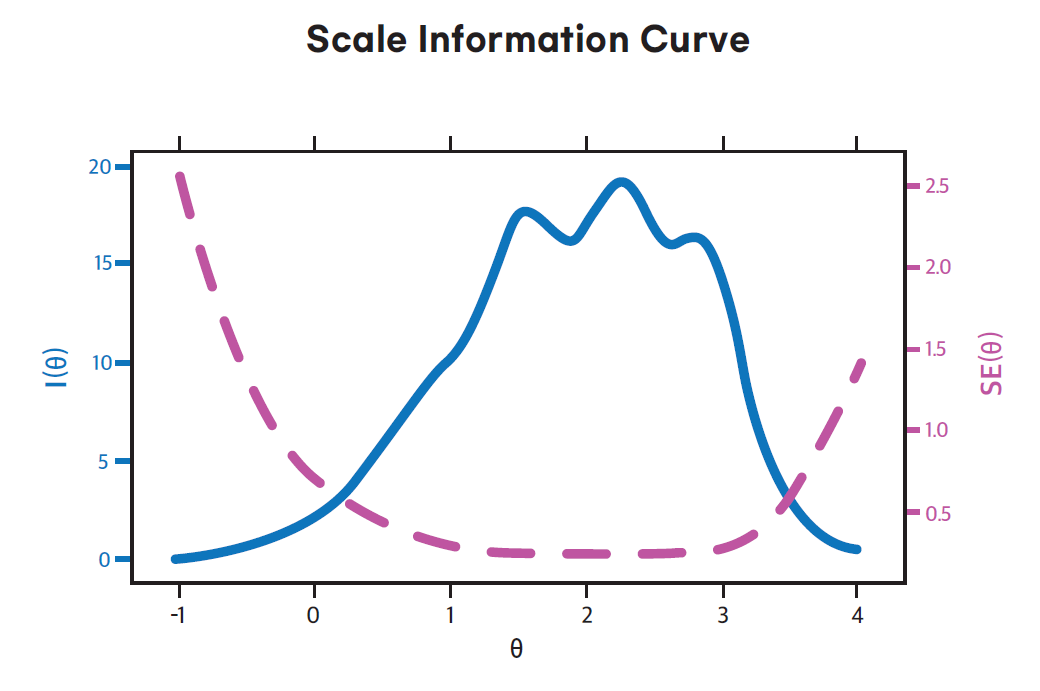
**

**Supplementary Figure 1. Scale information curve**
